# Five-year clinical follow-up of the STENTYS self-apposing stent in complex coronary anatomy: a single-centre experience with report of specific angiographic indications

**DOI:** 10.1007/s12471-018-1111-7

**Published:** 2018-04-13

**Authors:** H. Lu, R. J. Bekker, M. J. Grundeken, P. Woudstra, J. J. Wykrzykowska, J. G. P. Tijssen, R. J. de Winter, K. T. Koch

**Affiliations:** Department of Cardiology, Academic Medical Center, University of Amsterdam, Amsterdam, The Netherlands

**Keywords:** Stent designs, Complex lesions, Coronary artery disease

## Abstract

**Objectives:**

We sought to investigate angiographic indications for the use of the STENTYS technique and evaluated the long-term safety and clinical efficacy of the stent.

**Background:**

Coronary lesions involving complex anatomy, including aneurysmatic, ectatic, or tapered vessel segments often carry a substantial risk of stent malapposition. The self-apposing stent technique may reduce the risk of stent malapposition and therefore improve clinical outcomes.

**Methods:**

A total of 120 consecutive patients treated with the STENTYS stent were included (drug-eluting stent (DES) n = 101, bare-metal stent (BMS) n = 19). All lesions were scored for angiographic indications for the STENTYS stent, including aneurysms, ectasias, tapering, absolute diameters, bifurcation lesions, and saphenous vein grafts. Off-line quantitative coronary angiography analyses were performed pre-procedure and post-procedure. Five years follow-up was obtained including cardiac death, target vessel myocardial infarction (TV-MI), target vessel revascularisation, stent thrombosis, and the composite endpoint target vessel failure (cardiac death, TV-MI and target vessel revascularisation).

**Results:**

Angiographic indications for STENTYS use were aneurysm (30%), ectasia (19%), tapering (27%), bifurcation lesions (8%), and saphenous vein graft lesions (16%) and absolute diameters (22%). Mean maximal diameter was 4.51 ± 0.99 mm. At 5‑year follow-up target vessel failure rates were 24.1% in the total cohort (DES 22.8% vs. BMS 33%, *p* = 0.26). Definite stent thrombosis rate was 3.8% at 5‑year follow-up in this cohort with complex and high-risk lesions (DES 4.5% vs. BMS 0%, *p* = 0.39).

**Conclusions:**

Angiographic indications for the use of the self-apposing stent were complex lesions with atypical coronary anatomy. Our data showed reasonable stent thrombosis rates at 5‑year follow-up, considering the high-risk lesion characteristics.

**Electronic supplementary material:**

The online version of this article (10.1007/s12471-018-1111-7) contains supplementary material, which is available to authorized users.

## What’s new

- This is the first registry reporting on the indications that made interventionalists opt for the use of the STENTYS self-apposing technique in daily clinical practise with a 5-year clinical follow-up.
- This single-centre registry showed that operators tend to choose this stent technique in complex lesions, including coronary artery disease with aneurysm (30%), ectasia (19%), tapering (27%), bifurcation lesions (8%), saphenous vein graft lesions (16%) and absolute target vessel diameters (22%).
- At 5‑year follow-up, overall stent thrombosis rate was 3.8%, which is reasonable considering the complexity of this cohort where proper sizing with tubular balloon-expandable stents could be difficult in vessel segments with varying vessel diameters.
- The multi-centre enrolling SIZING registry will give us insights into the performance of this device in lesions with vessel diameter variance.

## Introduction

**Fig. 1:**
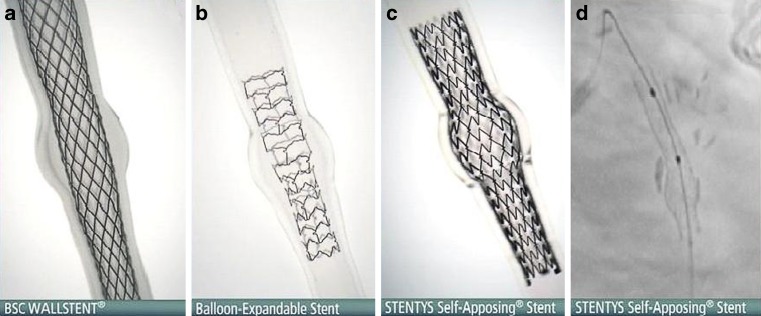
Stent apposition in a tapered aneurysmatic vessel. A tapered aneurysmatic tube which illustrates the stent apposition of the WALLSTENT (**a**), a balloon-expandable stent (**b**) the STENTYS self-apposing stent (**c**) and stent boost of the STENTYS stent in an aneurysmatic vessel in vivo (**d**)

Percutaneous coronary intervention of coronary lesions involving complex anatomy such as aneurysm, ectasia or tapering, remains challenging in daily clinical practice. Due to the varying vessel diameters within the target vessel, optimal stent sizing can be difficult. Particularly in these complex lesions, in-stent restenosis and late stent thrombosis remain significant problems, even when we use a contemporary drug-eluting stent (DES) [1]. When a stent/vessel size mismatch occurs, incompletely apposed stent struts could delay tissue coverage, and therefore predispose for the occurrence of stent thrombosis [2, 3]. Proper sizing using balloon-expandable stents in lesions with varying vessel diameters can be more difficult, since balloon-expandable stents are tubular by nature and limited to a maximum expansion diameter [4]. The nitinol self-apposing STENTYS stent (STENTYS SA, Paris, France) can adjust to varying lumen diameters (Fig. 1). This technique might improve clinical outcome after percutaneous coronary intervention of vessels involving varying diameters due to its superior strut apposition [5]. Moreover, large vessel diameters often exceed the expansion capacity of currently available balloon-expandable stents [6]. This stent can expand up to 6.0 mm which results in adequate apposition even in absolute vessel diameters. In the present single-centre study, we aimed to evaluate what the operator indication was for the use of the self-apposing stent in daily clinical practice. We report the angiographic indication for the stent, with angiographic and clinical outcomes up to 5‑year follow-up.

## Materials and methods

### Study patients and setting

All consecutive patients treated with the STENTYS stent since the introduction of the device in our centre from April 2010 until January 2016 were evaluated for this registry. Patients were excluded if they presented with ST-segment elevation myocardial infarction (STEMI) or were enrolled in a randomised trial. All other clinical indications for percutaneous coronary intervention were allowed. Percutaneous coronary intervention involving the STENTYS device was in the setting of routine clinical care. The choice for a drug-eluting stent or bare-metal stent (BMS) was at the discretion of the operator. Pre-implantation sizing was done by visual estimation. All patients provided written informed consent for this registry. All patients were pre-loaded with aspirin and a P2Y12 inhibitor, if not already on chronic therapy. Patients received 5,000 IU of unfractionated heparin at the start of the procedure. The use of peri-procedural glycoprotein IIb/IIIa receptor inhibitors was left at the discretion of the operator.

### Study device

The STENTYS stent is a new generation self-expanding device, made of a nitinol platform, a biocompatible nickel and titanium alloy. The stent is 6 French-compatible and is delivered using a rapid-exchange delivery system over a conventional 0.014″ guidewire. It is available in 3 lengths (17 mm, 22 mm and 27 mm), and in three diameter sizes: small (2.5–3.0 mm), medium (3.0–3.5 mm), and large (3.5–4.5 mm). The strut thickness is 102 microns (small size) or 133 microns (medium and large sizes). The large STENTYS can expand over 6.0 mm suitable for absolute vessel diameters of >4.5 mm. The stent is available as bare-metal stent and drug-eluting stent (paclitaxel or sirolimus). Since apposition is possible in large and variable vessel diameters of, for example, proximal and distal main branches, the stent can be used safely and effectively in bifurcation lesions [7–9] and in saphenous vein grafts [10]. Moreover, the nitinol platform can enlarge further after implantation. Therefore, the STENTYS device is extensively evaluated in patients with acute myocardial infarction in the APPOSITION trials [5, 11–13], which revealed lower rates of strut mal-apposition as compared with balloon-expandable stents and favourable clinical results. During the present study, the novel balloon-based delivery system Xposition was not yet available for commercial use.

### Angiographic data acquisition and definitions

An experienced interventional cardiologist (KTK) retrospectively reviewed the procedural report and procedural angiograms of all lesions treated with STENTYS to obtain lesion characteristics and to access the angiographic indication for this device if it was not stated in the report. The following angiographic indications were specified; aneurysm; ectasias; tapering; absolute reference vessel diameter 4.0–5.0 mm, bifurcation lesions and lesions located in saphenous vein grafts. Aneurysm was defined as localised or segmental dilatation which exceeds the diameter of normal adjacent segments by 1.5 times [14]. Ectasia was defined as irregular diffuse dilatation (>1.5 times the normal diameter) that involves more than one third of the length of the coronary artery [15, 16]. Tapering was defined as a significant diameter change of ≥1 mm from the proximal to the distal vessel segment. Offline quantitative coronary angiography (QCA) analyses were performed using dedicated software (QAngioXA version 7.3; Medis, Leiden, the Netherlands). Standardised QCA methodology was used including a bifurcation algorithm for bifurcation lesions. Pre- and post-procedural reference vessel diameter, minimal luminal diameter and percentage diameter stenosis (%DS) were obtained. Pre-implantation D‑max is obtained to assess maximal luminal diameters at baseline. Acute gain was defined as the difference between pre-procedural and post-procedural minimal luminal diameter. Longitudinal geographic mismatch on QCA was defined as the entire length of the lesion (as defined by QCA) not completely covered by the stent. Angiographic success was defined as final residual stenosis of less than 20% by offline QCA and thrombolysis in myocardial infarction (TIMI) 3 flow on the final angiogram without geographic mismatch.

### Follow-up and outcomes

Patients were contacted individually to obtain follow-up data. Hospital records and coronary angiograms were reviewed to complete information. Reported clinical outcomes included cardiac death, target vessel myocardial infarction (TV-MI), non-TV-MI, clinically indicated target lesion revascularisation, target vessel revascularisation (TVR), non-TVR, and definite/probable stent thrombosis according to the Academic Research Consortium definitions [17]. Target vessel failure was defined as the composite of cardiac death, TV-MI or TVR. Procedural success was defined as angiographic success without in-hospital target vessel failure.

### Statistical analysis

Continuous variables were presented as mean ± standard deviation, categorical variables as frequencies. We performed comparisons of variables using the two-sided Student-t test, chi-square or Fisher’s exact test, as appropriate. Event rates were assessed by Kaplan-Meier estimates and compared with the log-rank test. Follow-up was censored at 5 years or at the last known date of follow-up, whichever came first. We used the SPSS software package (version 24, IBM, Chicago, IL, USA).

## Results

### Baseline patient characteristics and procedural characteristics

Between April 2010 and January 2016, 120 patients were included in our registry including 19 STENTYS bare-metal stents, and 101 STENTYS drug-eluting stents. We report the outcome separately. Baseline clinical characteristics are summarised in Table 1 of the online supplementary material. Mean age was 65 ± 12 years in the group with a drug-eluting stents vs. 67 ± 13 in the group with a bare-metal stent. In the group with a drug-eluting stent, 46% of patients had stable angina, 16% unstable angina and 39% non-ST-segment elevation myocardial infarction (NSTEMI). In the group with a bare-metal stent, 53% of patients had stable angina, 26% unstable angina and 21% NSTEMI.

Lesion and procedural characteristics are shown in Table 2 of the online supplementary material. A total of 124 lesions were treated (study lesions). Of this total, 104 lesions were treated with the STENTYS drug-eluting stent and 20 with the STENTYS bare-metal stent. D‑max was 4.51 ± 0.99 mm in the group with a drug-eluting stent and 4.63 ± 1.05 mm in the group with a bare-metal stent (*p* = 0.64). A considerable number of patients had a pre-implantation D‑max of ≥4.0 to ≤5.0 mm (38% vs. 37% respectively, *p* = 0.95). In both groups, there was even a pre-implantation D‑max of ≥5.0 mm in approximately one third of cases (28% vs. 37% respectively, *p* = 0.42). Geographic mismatch occurred in 2% in the group with a drug-eluting stent vs. 5% in the group with a bare-metal stent (*p* = 0.46). Angiographic success was 69% in the group with a drug-eluting stent vs. 68% in the group with a bare-metal stent (*p* = 0.94), similar to the procedural success due to no in-hospital target vessel failure.

### Angiographic indications and results

**Fig. 2:**
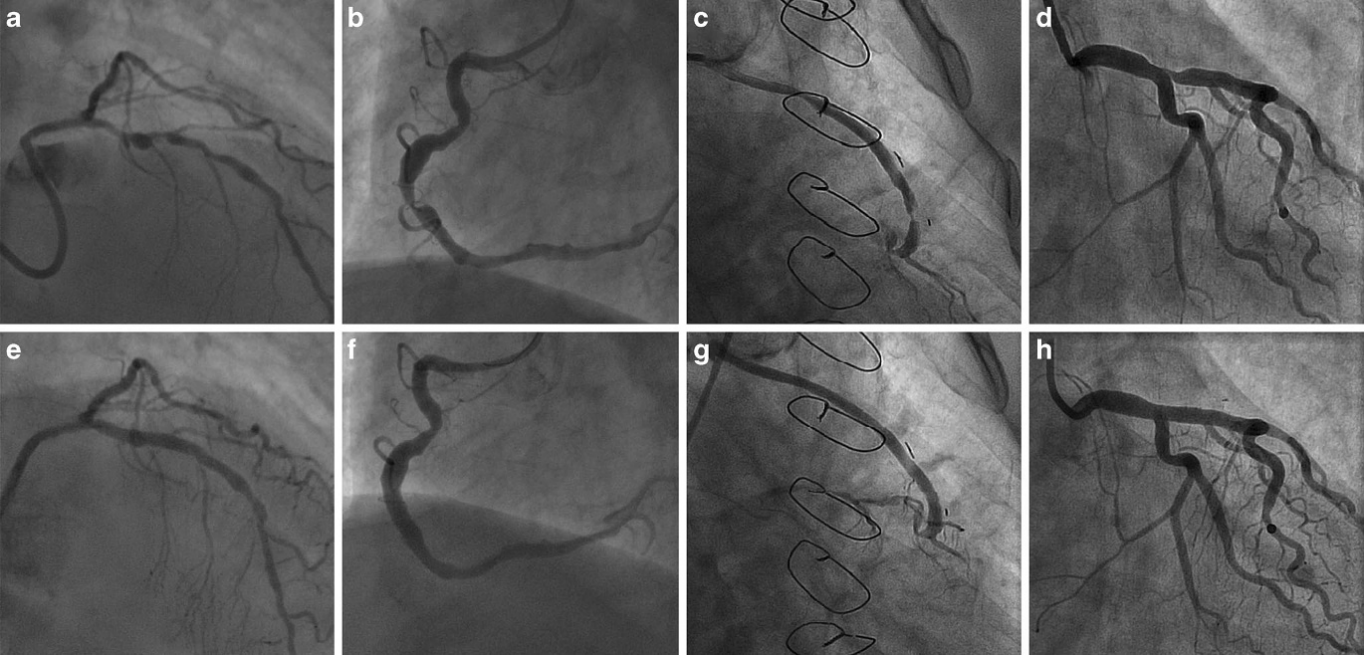
Examples of STENTYS cases. Pre-procedural and post-procedural coronary angiograms of 4 cases from our cohort including **a** pre-procedural angiogram of an aneurysmatic vessel, **e** result after STENTYS placement, **b** pre-procedural angiogram of an ectatic vessel, **f** result after STENTYS placement, **c** pre-procedural angiogram of saphenous vein graft lesion, **g** result after STENTYS placement, **d** pre-procedural angiogram of a tapered left main lesion, (H) result after STENTYS placement

Angiographic indications for operators to choose the STENTYS stent, were aneurysm (30%), ectasia (19%), tapering (27%), bifurcation lesions (8%), saphenous vein graft lesions (16%) and absolute target vessel diameters (22%) (Tab. 1). More than 1 angiographic indication could apply, for example, tapering and absolute target vessel diameter (Fig. 2). QCA results are summarised in Tab. 2. Reference vessel diameter, %DS and the minimal luminal diameter of all angiographic indications are shown separately illustrating the angiographic differences between the groups. In tapering, the reference vessel diameter of the proximal edge is remarkably larger than the reference vessel diameter in the distal edge of the treated segment. Angiographic outcomes for the bifurcation lesions are shown in Table 3 of the online supplementary material.

**Table 1:** Angiographic indications for STENTYS

Angiographic indications for STENTYS		DES	BMS	*p-value*
	*L* = 124	*L* = 104	*L* = 20	
Aneurysm	37 (30%)	31 (30%)	6 (30%)	0.99
Ectasia	24 (19%)	17 (16%)	7 (35%)	0.07
Tapering	33 (27%)	24 (23%)	9 (45%)	0.04
Diameters 4.0–5.0 mm	27 (22%)	21 (20%)	6 (30%)	0.38
Bifurcation lesion	10 (8%)	10 (10%)	0	0.36
Saphenous vein graft	19 (16%)	16 (16%)	3 (15%)	1

*N* (%), ≥1 indication may apply for 1 lesion

*DES* drug-eluting stent, *BMS* bare metal stent

**Table 2:** Pre-procedure and post-procedure quantitative coronary angiography (QCA) results

	All patients		Aneurysm & ectasia		Tapering		Absolute Diameters		Bifurcation (MB only)		SVG	
Parameter	Pre	Post	Pre	Post	Pre	Post	Pre	Post	Pre	Post	Pre	Post
RVD proximal edge (mm)	3.72 ± 0.85	3.87 ± 0.75	3.84 ± 0.85	4.02 ± 0.77	3.93 ± 1.01	3.97 ± 0.87	4.31 ± 0.97	4.34 ± 0.77	3.26 ± 0.44	3.37 ± 0.34	3.76 ± 0.99	3.97 ± 0.76
RVD in-stent (mm)	3.47 ± 0.84	3.56 ± 0.70	3.61 ± 0.82	3.73 ± 0.71	3.39 ± 0.87	3.53 ± 0.76	4.09 ± 0.93	4.11 ± 0.59	2.84 ± 0.45	2.95 ± 0.31	3.85 ± 1.00	3.98 ± 0.78
RVD distal edge (mm)	3.20 ± 1.01	3.33 ± 0.81	3.40 ± 1.11	3.55 ± 0.84	2.97 ± 0.93	3.10 ± 0.76	3.91 ± 0.81	3.93 ± 0.58	2.43 ± 0.60	2.63 ± 0.43	4.04 ± 1.01	3.96 ± 0.80
MLD in-stent (mm)	0.99 ± 0.58	2.90 ± 0.57	1.02 ± 0.54	3.00 ± 0.59	0.97 ± 0.36	2.86 ± 0.57	1.09 ± 0.73	3.30 ± 0.41	0.78 ± 0.32	2.47 ± 0.39	1.14 ± 0.84	3.20 ± 0.48
DS in-stent (%)	71.4 ± 13.5	18.2 ± 7.9	71.3 ± 14.7	19.7 ± 8.0	71.1 ± 8.8	18.5 ± 5.9	74.3 ± 13.0	19.3 ± 6.7	72.4 ± 10.4	16.5 ± 8.7	71.5 ± 17.1	18.2 ± 9.7
D-mean (mm)	3.06 ± 0.72	3.43 ± 0.62	3.15 ± 0.72	3.57 ± 0.62	3.03 ± 0.68	3.34 ± 0.64	3.56 ± 0.75	3.87 ± 0.53	2.55 ± 0.48	2.97 ± 0.32	3.56 ± 0.75	3.87 ± 0.53
D-max (mm)	4.53 ± 1.00		4.82 ± 1.08		4.60 ± 1.04		5.11 ± 0.89		3.99 ± 0.82		5.06 ± 1.10	
Lesion length (mm)	12.67 ± 7.40		14.33 ± 8.89		14.89 ± 8.38		14.23 ± 7.74		12.22 ± 5.50		11.90 ± 7.23	
Acute gain (mm)	1.95 ± 0.68		2.00 ± 0.67		1.93 ± 0.59		2.22 ± 0.69		1.74 ± 0.47		2.07 ± 0.92	

All values are expressed as mean ± standard deviation

*RVD* reference vessel diameter, *MLD* minimal lumen diameter, *DS* diameter stenosis, *D-max* maximum diameter, *D-mean* mean diameter, *MB* main branch, *SVG* saphenous vein graft

### Clinical outcomes

**Fig. 3:**
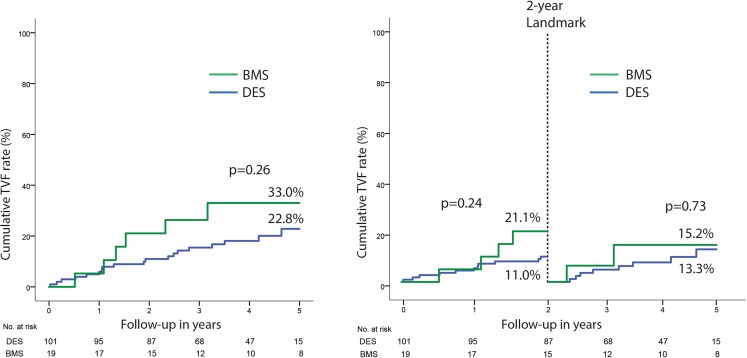
Kaplan-Meier estimates of target vessel failure. Cumulative event rate of the composite endpoint target vessel failure by Kaplan-Meier estimates with landmark analysis at 2‑year follow-up

**Fig. 4:**
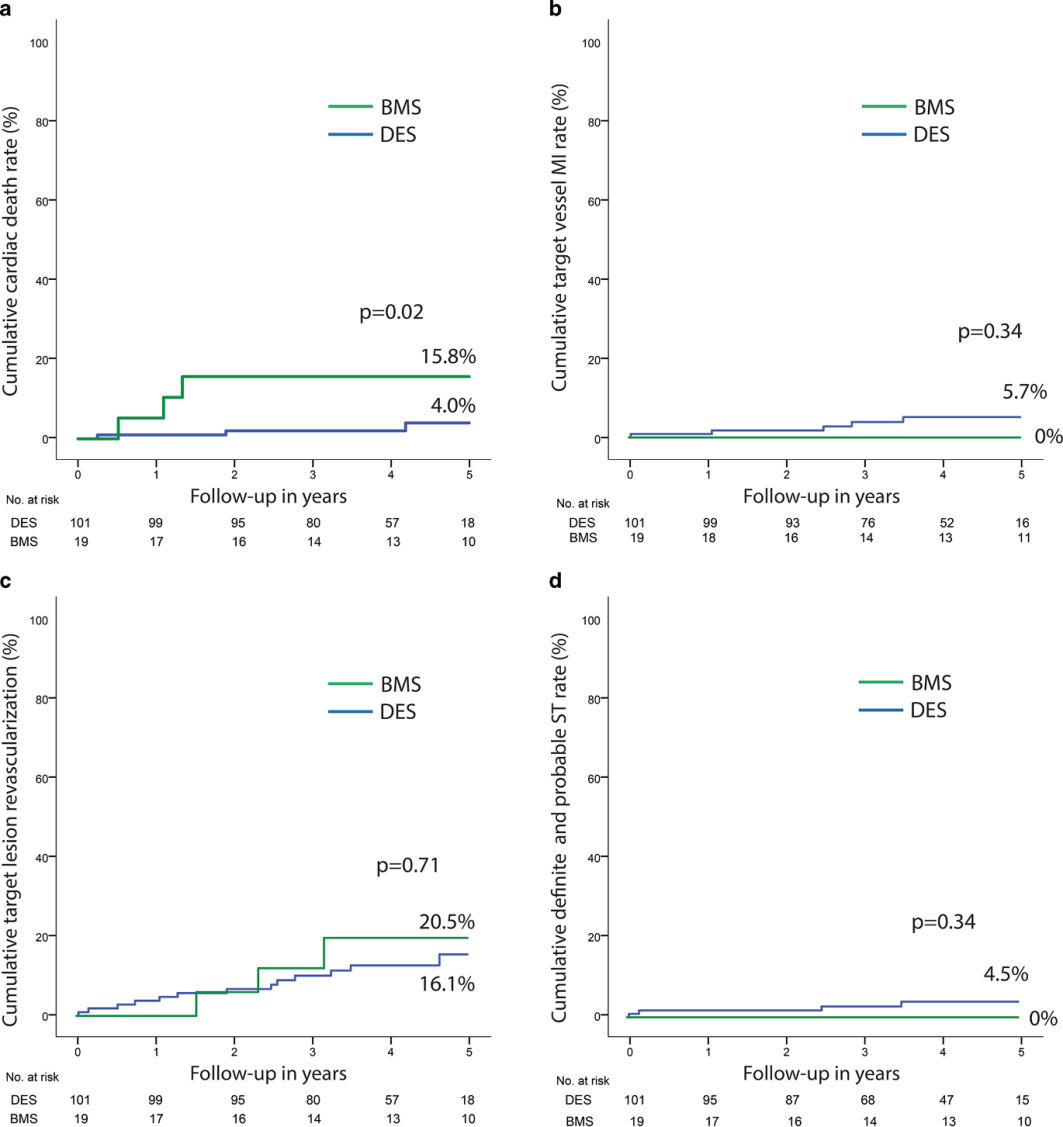
Kaplan-Meier estimates of cardiac death, target vessel myocardial infarction, target lesion revascularisation, and probable and definite stent thrombosis. **a** Cumulative event rate of individual endpoint cardiac death by Kaplan-Meier estimates. **b** Cumulative event rate of the individual endpoint target vessel myocardial infarction by Kaplan-Meier estimates. **c** Cumulative event rate of the individual endpoint target lesion revascularisation by Kaplan-Meier estimates. **d** Cumulative event rate of the individual endpoint probable and definite stent thrombosis by Kaplan Meier estimates

Clinical follow-up was obtained for all patients with a median follow-up of 51 months (IQR 42–60). One patient was lost to follow-up due to emigration to Suriname 2 years after baseline percutaneous coronary intervention and is censored from the date of emigration. Target vessel failure was observed in 25 patients (24.1%), 19 (22.8%) in the group with a drug-eluting stent vs. 6 (33%) in the group with a bare-metal stent (Tab. 3). Landmark analysis at 2‑year up to 5‑year follow-up revealed an incremental target vessel failure rate of 13.3% in the group with a drug-eluting stent and 15.2% in the group with a bare-metal stent (*p* = 0.73) (Fig. 3). In the total cohort, definite stent thrombosis occurred in 4 cases (3.8%). Stent thrombosis characteristics are shown in Table 4 of the online supplementary material. Individual clinical endpoints are shown in Tab. 3 and Fig. 4. Clinical outcomes per angiographic indication are shown in Table 5 of the online supplementary material. Comparison between the bare-metal stent group and the drug-eluting stent group should be interpreted with caution due to the non-randomised design and the small sample size.

**Table 3:** Patient event rates at 5‑year follow-up

Clinical event	Total		DES		BMS		*p*-value
	(*n* = 120)		(*n* = 101)		(*n* = 19)		
	*n*	Event rate	*n*	Event rate	*n*	Event rate	
*Target vessel failure*							
Cardiac death, TV-MI and TVR	25	24.1%	19	22.8%	6	33.0%	0.26
*Target Vessel Failure between 2 and 5 years*							
Cardiac death, TV-MI and TVR	10	13.1%	8	13.3%	2	15.2%	0.73
*Other composite endpoints*							
Cardiac death and TV-MI	11	10.5%	8	9.7%	3	15.8%	0.31
Cardiac death, TV-MI and TLR	22	21.7%	16	20.0%	6	33.0%	0.13
*Components of composite endpoints*							
Cardiac death	6	5.7%	3	4.0%	3	15.8%	0.02
TV-MI	5	4.9%	5	5.7%	0	0.0%	0.34
TVR	19	19.0%	16	19.2%	3	20.5%	0.98
*Stent thrombosis*							
Definite	4	3.8%	4	4.5%	0	0.0%	0.39
Probable	1	1.6%	1	2.0%	0	0.0%	0.61
*Other events*							
Clinically indicated TLR	16	16.4%	13	16.1%	3	20.5%	0.71
Non-cardiac death	3	3.2%	3	3.8%	0	0.0%	0.46
MI not related to target vessel	2	1.9%	2	2.2%	0	0,0%	0.55
Non-TVR	15	13.3%	15	15.7%	0	0.0%	0.09

Values are *n* (number of patients) with event rates calculated using the Kaplan-Meier method

*BMS* bare-metal stent, *DES* drug-eluting stent, *MI* myocardial infarction, *TV-MI* target vessel myocardial infarction, *TVR* target vessel revascularisation, *TLR* target lesion revascularisation

## Discussion

This study evaluates the angiographic indications for the use of the STENTYS device according to experienced interventionalists with the report of 5‑year clinical outcome in a subgroup of complex patients. Indications for STENTYS use was coronary aneurysm, ectasia, tapering, absolute vessel diameters, bifurcation lesions and lesions located in saphenous vein grafts. Our QCA data demonstrates that this device is effective in large variable reference vessel diameters in a cohort with high-risk complex coronary anatomy.

### Clinical experience with the STENTYS stent

Several theoretic advantages of the nitinol self-apposing platform were previously evaluated in selected cohorts. Naber et al. observed a definite stent thrombosis rate of 1% at 6‑month and 0% at 12-month follow-up in bifurcation lesions [7]. A large STEMI registry with STENTYS bare-metal stent and paclitaxel-eluting stent (PES) revealed definite stent thrombosis rates of 3.3% at 2‑year follow-up [13]. Less data is available for the stent performance in all-comer populations. An evaluation of a small real-world cohort reveals that interventionalists chose the STENTYS device in a comparable selection of angiographic situations, including bifurcation lesions, acute coronary syndrome and ectatic coronaries, with a stent thrombosis rate of 2.5% at 21 ± 13 months [18]. Another real-world single-centre experience with both STENTYS bare-metal stent and paclitaxel-eluting stent from Italy reported a stent thrombosis rate of 1.8% at 23.6 ± 12.6 months [19]. A larger multicentre cohort reported stent thrombosis rates of 2.6% at 2.5 years in all-comer patients, based on usage of STENTYS bare-metal stent, paclitaxel-eluting stent and sirolimus-eluting stent [20]. In the present study, we observed a geographic mismatch in 2% in the group with a drug-eluting stent and 5% in the group with a bare-metal stent (*p* = 0.46), and overall stent thrombosis rate of 3.8% at 5‑year follow-up. Considering the complexity of our cohort including both STENTYS bare-metal stent and paclitaxel-eluting stent, these indicators for device performance are acceptable. The low angiographic success rates of 69% in the group with a drug-eluting stent and 68% in the group with a bare-metal stent is partly explained by the fact the we incorporated a geographic mismatch and measured the residual stenosis on off-line QCA. These success rates should be interpreted with caution since QCA analyses might overestimate residual stenosis in aneurysmatic and ectatic lesions.

### Comparison with contemporary balloon expandable stents

It is well-known that complex target lesion anatomy is associated with an increased risk of adverse outcome [21, 22]. Using the SYNTAX score, an angiographic scoring system quantifying the complexity of coronary artery disease, it is possible to identify high-risk patients based on angiographic characteristics [23, 24]. Unprotected left main, multi-vessel disease, longer lesions, bifurcation or trifurcation lesions and large thrombus load are incorporated and considered more complex. A recent pooled analysis evaluating new-generation balloon-expandable drug-eluting stents, including stents eluting everolimus and zotarolimus, and biodegradable polymer stents eluting biolimus and sirolimus, demonstrated a definite stent thrombosis rate of 1.0% at 2‑year follow-up in patients with a higher coronary complexity (SYNTAX score > 11) [25]. The RESOLUTE all-comers trial comparing the Resolute zotarolimus-eluting stent with the Xience V everolimus-eluting stent revealed definite stent thrombosis rates of 1.2% vs. 0.4% (*p* = 0.14) respectively in a pre-specified subgroup of complex patients at 1‑year follow-up [26]. The same trial reported definite stent thrombosis rates of 1.6% vs. 0.8% (*p* = 0.08) respectively at 5‑year follow-up in an all-comers population [27]. Our observations of much higher stent thrombosis rates could partially be explained by the atypical complexities including aneurysms, ectasia and large tapering. Moreover, the patients were treated with the STENTYS bare-metal stent or paclitaxel-eluting stent delivered with the conventional delivery system. The Xposition delivery system with the sirolimus-eluting STENTYS device, where precise stent delivery is made possible by a delivery balloon which is retracted after stent delivery, might improve clinical outcome in complex coronary anatomy [28].

### Study limitations

This study is limited by its observational design. It is a small, single-centre cohort study of complex lesions. A matched control group with such atypical anatomical high-risk lesions treated with balloon-expandable stents was not available. The STENTYS use, including the choice for STENTYS drug-eluting stent or bare-metal stent was at the discretion of the operator. The angiograms were reviewed by a single expert only. No routine angiographic follow-up was performed in these patients. Finally, clinical outcomes were not adjudicated by an independent clinical endpoint committee.

## Conclusion

This single-centre registry showed that operators tend to choose this stent technique in complex lesions. Considering the high-risk lesion characteristics, stent thrombosis rates are reasonable at 5‑year follow-up. The STENTYS platform seems safe and effective in patients with an atypical anatomy. The enrolling SIZING registry will give us insights into the performance of this device in lesions with vessel diameter variance [29].

## Caption Electronic Supplementary Material

Fig. 1 IVUS post-stenting showing apposition of the STENTYS stent in a tapered left main coronary artery. The proximal section of the stent, which was located in the left main coronary artery, has a larger diameter (4.4 mm) and tapers to the distal section of the stent, located in the left ascending descending coronary artery, with a diameter of 2.6 mm (right panel), with good overall stent apposition in both vessel segments

Table 1. Baseline clinical characteristics
Table 2. Lesion and procedural characteristics
Table 3. Quantitative coronary angiography results (QCA) Bifurcation table
Table 4. Characteristics of patients with stent thrombosis
Table 5. Clinical outcomes by angiographic indication
