# Supplementary figures and images for: Five-year clinical follow-up of the STENTYS self-apposing stent in complex coronary anatomy: a single-centre experience with report of specific angiographic indications
Source: Neth Heart J. 2018 Apr 13;26(5):263–71. doi: 10.1007/s12471-018-1111-7 (PMC5910317; doi:10.1007/s12471-018-1111-7)

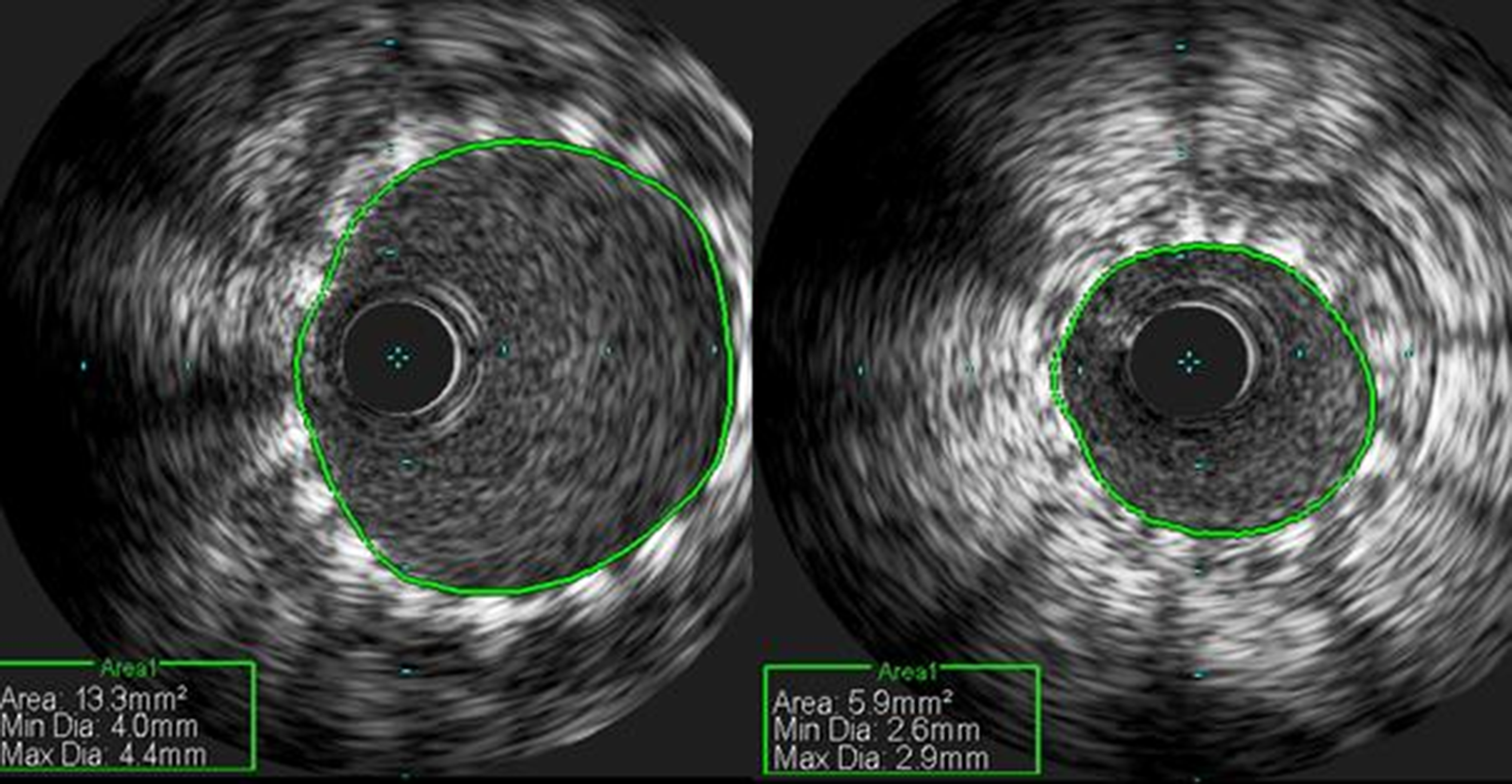

Supplement: Supplementary file 1 — Fig. 1 IVUS post-stenting showing apposition of the STENTYS stent in a tapered left main coronary artery. The proximal section of the stent, which was located in the left main coronary artery, has a larger diameter (4.4 mm) and tapers to the distal section of the stent, located in the left ascending descending coronary artery, with a diameter of 2.6 mm (right panel), with good overall stent apposition in both vessel segments [file 12471_2018_1111_MOESM1_ESM.tif]
