# Supplementary material for: Five-year clinical follow-up of the STENTYS self-apposing stent in complex coronary anatomy: a single-centre experience with report of specific angiographic indications
Source: Neth Heart J. 2018 Apr 13;26(5):263–71. doi: 10.1007/s12471-018-1111-7 (PMC5910317; doi:10.1007/s12471-018-1111-7)
Supplement: Supplementary file 2 — Table 1. Baseline clinical characteristics Table 2. Lesion and procedural characteristics Table 3. Quantitative coronary angiography results (QCA) Bifurcation table Table 4. Characteristics of patients with stent thrombosis Table 5. Clinical outcomes by angiographic indication [file 12471_2018_1111_MOESM2_ESM.doc]

| **Online supplement**  **Table 1 Baseline clinical characteristics** | | |  |  |  |
| --- | --- | --- | --- | --- | --- |
| **Characteristic** | | DES n = 101 | BMS n = 19 | *p-value* |  |
| Demographics | |  |  |  |  |
|  | Age | 65 ± 12 | 67 ± 13 | 0.56 |  |
|  | Male gender | 81 (80%) | 13 (68%) | 0.25 |  |
| Risk factors for CAD | |  |  |  |  |
|  | Diabetes | 17 (17%) | 3 (16%) | 0.89 |  |
|  | Hypertension | 44 (44%) | 13 (68%) | 0.06 |  |
|  | Hypercholesterolemia | 29 (30%) | 7 (37%) | 0.53 |  |
|  | Current smoker | 31 (31%) | 6 (32%) | 0.98 |  |
|  | Family history of CAD | 49 (50%) | 6 (32%) | 0.14 |  |
|  | Renal dysfunction | 5 (5%) | 4 (21%) | 0.02 |  |
| History | |  |  |  |  |
|  | Previous MI | 21 (21%) | 4 (21%) | 0.,99 |  |
|  | Previous PCI | 25 (25%) | 5 (26%) | 0.9 |  |
|  | Previous CABG | 19 (19%) | 4 (21%) | 0.84 |  |
|  | Stroke | 10 (10%) | 2 (11%) | 0.94 |  |
|  | Congestive heart failure | 3 (3%) | 0 (0%) | 0.44 |  |
| Clinical indications for PCI | |  |  |  |  |
|  | Stable angina | 46 (45%) | 10 (53%) | 0.57 |  |
|  | Unstable angina | 16 (16%) | 5 (26%)) | 0.27 |  |
|  | Non STEMI | 39 (39%) | 4 (21%) | 0.14 |  |
|  |  |  |  |  |  |
| Values are *n* (%) or mean ± SD, *CAD* coronary artery disease, *MI* myocardial infarction, | | | | |  |
| *PCI* percutaneous coronary intervention, *CABG* coronary artery bypass grafting, | | | | |  |
| *STEMI* ST-segment elevation myocardial infarction | | | | |  |

**Online supplement**

**Table 2. Lesion and procedural characteristics**

| **Lesion and procedural characteristics** | | | **DES** | | **BMS** | | *p-value* |  |
| --- | --- | --- | --- | --- | --- | --- | --- | --- |
|  |  |  |  |  |  |  |  |  |
| Study lesions treated with STENTYS | | | **(L = 104)** | | **(L = 20)** | |  |  |
| *Location of study lesion* | |  |  |  |  |  |  |  |
|  | Left main |  | 7 | 7% | 0 | | 0.59 |  |
|  | LAD |  | 27 | 26% | 7 | 35% | 0.41 |  |
|  | RCx |  | 19 | 18% | 2 | 10% | 0.52 |  |
|  | RCA |  | 35 | 34% | 8 | 40% | 0.59 |  |
|  | SVG |  | 16 | 15% | 3 | 15% | 1 |  |
| *ACC/AHA lesion classification* | |  |  |  |  |  |  |  |
|  | A |  | 9 | 9% | 0 | | 0.35 |  |
|  | B1 |  | 20 | 19% | 5 | 25% | 0.55 |  |
|  | B2 |  | 53 | 51% | 9 | 45% | 0.63 |  |
|  | C |  | 22 | 21% | 6 | 30% | 0.39 |  |
| Second stent placement at target lesion | | | 18 | 17% | 4 | 20% | 0.75 |  |
| Stent length (mm) | | 17mm | 2 | 2% | 0 | | 0.53 |  |
|  |  | 22mm | 46 | 44% | 14 | 70% | 0.04 |  |
|  |  | 27mm | 56 | 54% | 6 | 30% | 0.05 |  |
| Stent diameter size (mm) | | 2.5-3.0 | 3 | 3% | 1 | 5% | 0.51 |  |
|  |  | 3.0-3.5 | 36 | 35% | 7 | 35% | 0.97 |  |
|  |  | 3.5-4.5 | 65 | 63% | 12 | 60% | 0.83 |  |
| Direct stenting | |  | 12 | 12% | 5 | 25% | 0.15 |  |
| Post dilatation performed | |  | 95 | 92% | 15 | 75% | 0.04 |  |
| Max pressure per lesion (atmosphere) | | | 13,9 ± 4,1 | | 14,8 ± 4,2 | | 0.5 |  |
| Use of distal embolization protection device | | | 10 | 10% | 2 | 10% | 1 |  |
| *Postprocedure TIMI flow* | | Grade 0 | 0 | | 0 | | ­ |  |
|  |  | Grade 1 | 0 | | 0 | | ­ |  |
|  |  | Grade 2 | 3 | 3% | 1 | 5% | 0.51 |  |
|  |  | Grade 3 | 101 | 97% | 19 | 95% | 0.51 |  |
| Angiographic success | |  | 70 | 69% | 13 | 68% | 0.94 |  |
| Procedural success | |  | 70 | 69% | 13 | 68% | 0.94 |  |
| *QCA measurement* | |  |  |  |  |  |  |  |
|  | Pre-implantation Dmax (mm) |  | 4,51 ± 0,99 | | 4,63 ± 1,05 | | 0.64 |  |
|  | Pre-implantation Dmax ≥4.0 to ≤ 5.0 mm (%) | | 38 | 38% | 7 | 37% | 0.95 |  |
|  | Pre-implantation Dmax ≥5.0 mm (%) | | 28 | 28% | 7 | 37% | 0.42 |  |
|  | Residual stenosis >20% on QCA, n (%) | | 28 | 28% | 5 | 26% | 0.9 |  |
|  | Longitudinal geographic mismatch, n (%) | | 2 | 2% | 1 | 5% | 0.46 |  |
|  |  |  |  |  |  |  |  |  |
| Values are *n* (%) or mean ± SD, *PCI* percutaneous coronary intervention, *LAD* left anterior descending, *RCx* ramus | | | | | | | |  |
| circumflexus, *RCA* right coronary artery, *SVG* saphenous vein graft, *TIMI* thrombolysis in myocardial infarction, | | | | | | | | |
| *QCA* quantitative coronary angiography, *Dmax* maximal luminal diameter | | | | | | | |  |

**Online supplement**

**Table 3 Quantitative coronary angiography results (QCA) Bifurcation table**

|  | RVD (mm) | | DS (%) | | MLD (mm) | | Acute gain (mm) |
| --- | --- | --- | --- | --- | --- | --- | --- |
| Parameter | Pre | Post | Pre | Post | Pre | Post |
| 5 mm prox edge | 3.48±0.99 | 3.82±0.98 | 6.37±6.55 | 13.66±5.72 | 3.25±0.98 | 3.28±0.80 | 0.03±0.48 |
| PMB in-stent | 3.36±1.10 | 4.05±1.14 | 28.73±22.43 | 13.96±9.52 | 1.60±1.18 | 2.82±0.62 | 1.21±0.89 |
| DMB in-stent | 2.80±0.75 | 2.99±0.65 | 65.24±10.05 | 15.10±6.68 | 0.96±0.40 | 2.48±0.62 | 1.52±0.52 |
| 5 mm distal edge | 2.40±0.57 | 2.70±0.59 | 20.10±21.40 | 21.10±16.48 | 1.97±0.82 | 2.13±0.71 | 0.16±0.43 |
| SB | 2.10±0.54 | 2.28±0.68 | 39.11±19.84 | 23.79±11.71 | 1.31±0.72 | 1.77±0.73 | 0.45±0.68 |
| All values are expressed as mean ±standard deviation. PMB; proximal main branch, DMB; distal main branch, SB; side branch, RVD; reference vessel diameter, DS; diameter stenosis, MLD; minimal lumen diameter. | | | | | | | |

**Online supplement**

**Table 4 Characteristics of patients with stent thrombosis**

| Patient | Stent thrombosis type | Stent type | Days | Lesion location | DAT at time of event | Outcome | Repeat revascularization |
| --- | --- | --- | --- | --- | --- | --- | --- |
| 82-year-old male | Definite | DES | 9 | LAD/D1 | Yes | MI | Yes, balloon angioplasty |
| 44-year-old male | Definite | DES | 1280 | RCX | Yes | MI | Yes, stent placement |
| 67-year-old male | Definite | DES | 56 | RCX | Yes | no MI | Yes, balloon angioplasty |
| 80-year-old female | Definite | DES | 908 | RCA | Yes | MI | Yes, balloon angioplasty |
| 77-year-old male | Probable | DES | 1528 | NA | Unknown | Unknown cause of death | NA |
| DAT dual antiplatelet therapy MI myocardial infarction NA not applicable | | | | | | | |

**Online supplement**

**Table 5 Clinical outcomes by angiographic indication**

| **Outcomes by indication** | | | Aneurysm | | Ectasia | | Tapering | | Diameters 4,0 - 5,0 mm | | Bifurcation lesion | | SVG | |
| --- | --- | --- | --- | --- | --- | --- | --- | --- | --- | --- | --- | --- | --- | --- |
|  |  |  | n = 37 | | n = 24 | | n = 33 | | n = 27 | | n = 10 | | n = 19 | |
|  |  |  | *n.* |  | *n.* |  | *n.* |  | *n.* |  | *n.* |  | *n.* |  |
|  |  |  |  | event rate |  | event rate |  | event rate |  | event rate |  | event rate |  | event rate |
| Target Vessel Failure | | | 6 | 20.9% | 5 | 22.6% | 9 | 29.7% | 4 | 15.2% | 2 | 22.9% | 8 | 51.1% |
|  | Cardiac death | | 2 | 8.1% | 1 | 4.3% | 1 | 3.% | 2 | 7.6% | 0 | 0% | 2 | 10.5% |
|  | TV-MI |  | 2 | 6.8% | 1 | 5.3% | 2 | 7.3% | 0 | 0% | 1 | 10% | 0 | 0% |
|  | TVR |  | 4 | 13.3% | 4 | 18.9% | 8 | 27.4% | 2 | 8.1% | 2 | 22.9% | 6 | 45% |
| Other events | |  |  |  |  |  |  |  |  |  |  |  |  |  |
|  | Clinically indicated TLR | | 4 | 13.3% | 4 | 18.9% | 6 | 20.7% | 1 | 4.5% | 2 | 22.9% | 5 | 41.5% |
|  | Definite Stent thrombosis | | 2 | 6.8% | 0 | 0% | 2 | 7.3% | 0 | 0% | 0 | 0% | 0 | 0% |
|  | Probable Stent thrombosis | | 1 | 5.6% | 0 | 0% | 0 | 0% | 0 | 0% | 0 | 0% | 0 | 0% |
|  | Non-TVR |  | 6 | 17.4% | 3 | 13.3% | 3 | 10.4% | 4 | 14.8% | 0 | 0% | 4 | 24.8% |
|  |  |  |  |  |  |  |  |  |  |  |  |  |  |  |
| Values are *n* (number of patients) with event rates calculated using the KM-method. TV-MI Target Vessel Myocardial infarction | | | | | | | | | | | | |  |  |
| TVR target vessel revascularization, TLR target lesion revascularization | | | | | | | | | | | | | | |
